# An improved method for the precise unravelment of non-shivering brown fat thermokinetics

**DOI:** 10.1038/s41598-021-84200-1

**Published:** 2021-02-26

**Authors:** Rebecca Oelkrug, Jens Mittag

**Affiliations:** Institute for Endocrinology and Diabetes – Molecular Endocrinology, Center of Brain Behavior and Metabolism CBBM, University of Lübeck, Ratzeburger Allee 160, 23562 Lübeck, Germany

**Keywords:** Fat metabolism, Animal physiology

## Abstract

Since the discovery of functional brown adipose tissue (BAT) in adult humans, research on BAT gained a new popularity to combat obesity and related comorbidities. To date, however, methods to quantify BAT thermogenesis are often either highly invasive, require advanced equipment, are time consuming or of limited sensitivity. Here we present a simple yet highly effective and minimally invasive protocol for the Precise Unravelment of Non-shivering brown fat thermoKinetics (PUNK) in mice using infrared thermography in combination with Vaseline to brush up the fur between the shoulder blades. We also use physiological and molecular readouts including indirect calorimetry, qPCR and Western Blots to test our protocol. Our study demonstrates that Vaseline before thermography vastly advances the reproducibility and quality of BAT infrared pictures or videos, as it exposes the skin above the BAT for a direct line of sight for the infrared camera and thereby circumvents the well-known problems associated with shaving and anaesthesia. We subsequently validate that this approach does not affect physiological and molecular BAT function, but instead leads to more robust and less variable results when comparing for instance norepinephrine stimulation tests or knockout animals. Taken together, the PUNK protocol for BAT thermography quickly and effectively improves scientific outcomes of this method, and can be easily added to existing paradigms. Consequently, it safes money, time and experimental animals, thereby putting the 3R’s principles of animal welfare into practice.

## Introduction

Active brown adipose tissue (BAT) is currently discussed as an important target to combat obesity and metabolic dysfunction, as it burns extra calories as heat and extracts glucose as well as fatty acids from the blood stream to fuel cellular metabolism^1–3^. Although the uncoupling protein 1 (UCP1) is crucial for BAT function, several recent studies have shown that the magnitude of *Ucp1* mRNA expression is not a suitable read-out to estimate BAT or beige fat activity^4,5^, highlighting the need for reliable in vivo techniques to quantify BAT thermogenesis in a physiologically meaningful manner.

Infrared (IR) thermography is a method that directly measures BAT heat production and subsequently UCP1-mediated uncoupling of the mitochondrial respiratory chain in vivo^6^, whereas other techniques that are commonly used to estimate BAT activity, like PET-CTs, fMRIs or ^13^C-MRS scans, determine BAT non-shivering thermokinetics indirectly from BAT metabolism or blood flow (reviewed by^7^). Here, the animals need to be anaesthetised and are exposed to potentially harmful radiation during these measurements, which makes it difficult, if not impossible, to conduct longitudinal studies. Infrared thermography has been successfully used in many rodent studies to directly measure BAT thermogenesis^5,8–10^, however there is an ongoing debate on whether to anesthetize the animals and/or remove their fur during the measurements to gain more reproducible results^6,11–14^. These procedures not only increase the burden for the animals but in case of anaesthetised animals lead to an underestimation of the maximum BAT temperature, which reduces the quality of scientific outcomes. Moreover, shaving can induce stress and inflammation in the affected skin areas and alters the conductivity of the area covering the BAT, thereby potentially inducing a thermogenic gene program in subcutaneous white and beige adipocytes in the underlying BAT area^15^. Consequently, a reliable and minimally invasive protocol to quantify BAT thermogenesis in rodents is urgently needed, which avoids the problems associated with shaving or anaesthesia.

Here we present a new protocol for the Precise Unravelment of Non-shivering brown fat thermoKinetics (PUNK), which eliminates of the necessity of using anesthetics or shaving in infrared thermography, and leads to more stable and conclusive scientific results.

## Material and methods

### Animal husbandry

Wild-type male C57BL/6NCr mice were purchased from Charles River and homozygous male UCP1 knockout (UCP1-KO) and wild-type (WT) littermates (genetic background C57BL/6 J) were obtained from our breeding colony at the animal facility of the University of Lübeck. Animals (3–5 months) were single housed on a constant 12-h light/12-h dark cycle at 23 ± 1 °C and cages were equipped with cotton nestlets. Unless otherwise stated animals had free access to food and water (fortified breeding diet #1314 from Altromin, Germany). All procedures were approved by the MELUND Schleswig-Holstein (Germany), and all methods were carried out in accordance with relevant guidelines and regulations. The required sample size was calculated from previous studies using G*Power version 3.1.9.4. (University of Düsseldorf, Germany), with power (1 − β) set to 0.80, and a significance level α of 0.05. No inclusion or exclusion criteria were set, animals were randomly assigned to groups and RO was aware of the group allocation.

### Infrared thermography and rectal body temperature

**Figure 1:**
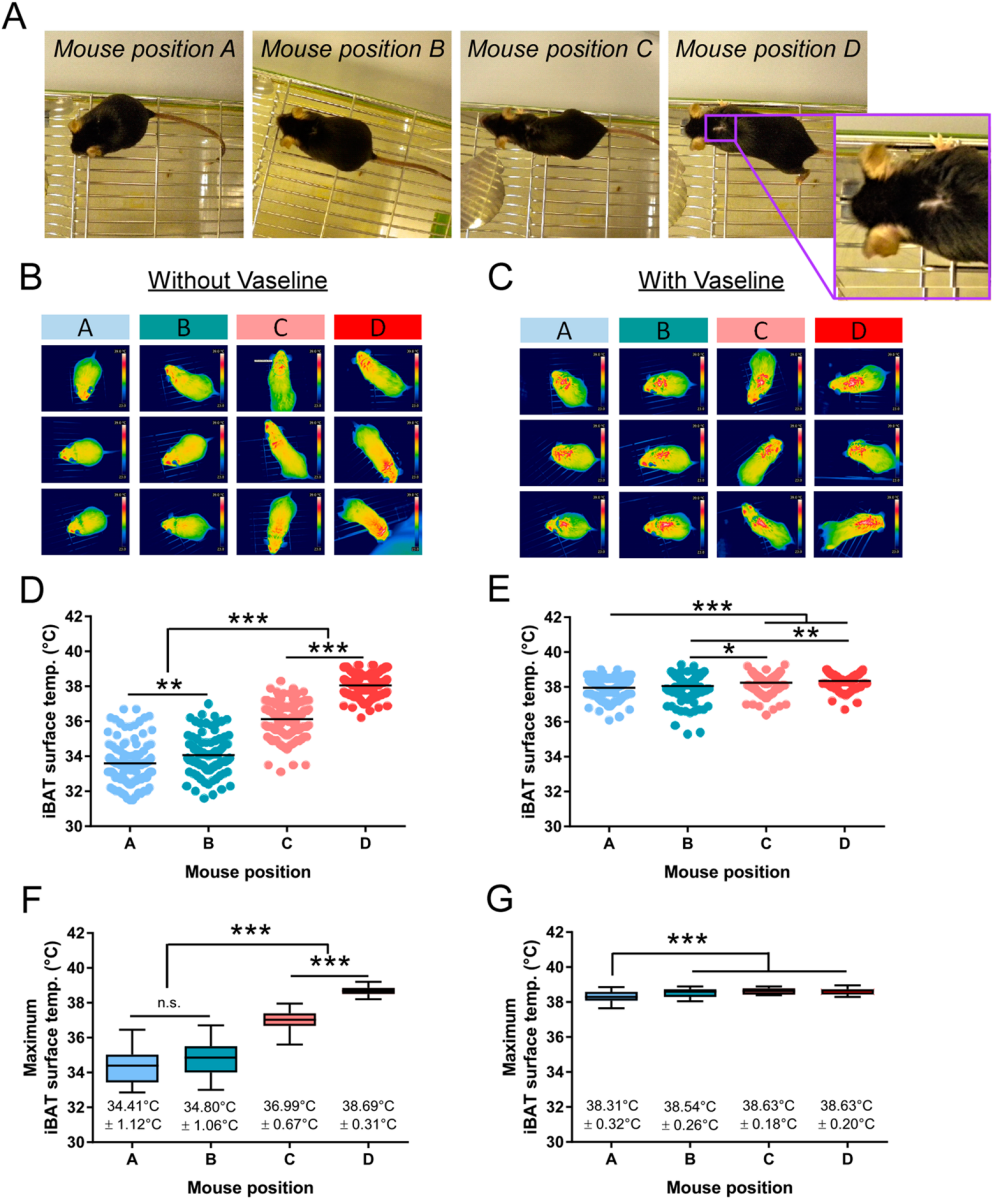
Vaseline enables more accurate and reproducible measurements of iBAT temperature using infrared (IR) thermography. (**A**) Mice were categorized according to their posture/position. Mouse position A: The mouse sits curled up or is grooming itself; no direct view on the skin. Mouse position B: The animal is moving or stretches forward a little; no direct view on the skin. Mouse position C: The animal stretches forward, but looks upward; no direct view on the skin. Mouse position D (including a closeup picture of the fur): The animal stretches forward and looks down; direct view on the skin. (**B**,**C**) Representative infrared pictures of three different mice per position without and with Vaseline. (**D**,**E**) iBAT surface temperature of all infrared pictures taken within two sessions (3 min per day) of the same animals without and with Vaseline (N = 12, n = 108–147 (without Vaseline), n = 118–165 (with Vaseline). (**F**,G) Mean of maximum iBAT temperature of the two sessions presented in (**D**,**E**), revealing more stable measurements after Vaseline application (N = 12, n = 24). Data are presented as mean (**D**,**E**) or box plot (min. to max. (**F**,**G**). *p < 0.05, **p < 0.01, ***p < 0.001 (1-way ANOVA with Tukey’s post-hoc test (**D**,**E**) and 1-way repeated measures ANOVA with Tukey’s post-hoc test (**F**,**G**)).

Infrared pictures were taken with a hand-held camera (T335, with a thermal sensitivity of < 0.05 °C and an accuracy of 2% max 2 °C, FLIR, Sweden) on freely moving animals to minimize stress [N = 12 (Fig. 1) and N = 7 (UCP1-KO versus WT, Fig. 4A,B)]. Therefore, animals were placed on their cage grid for max. 3 min and pictures were taken from the interscapular brown adipose tissue (iBAT) area (between the shoulder blades) at the same time every day (9:30 a.m.–10:00 a.m.). After one training day, iBAT temperature was measured of animals without (−) Vaseline on two consecutive days. On the third and fourth experimental day, a small amount of Vaseline (~ 10 mg, Balea/DM, Germany) was applied with a sterile cotton swab (#974117, Nobamed, Germany) between the shoulder blades of the animals to brush up the neck fur before the pictures were taken (with ( +) Vaseline, see closeup picture of the fur provided in Fig. 1A). Therefore, animals were placed on the cage lid or briefly held by the tail. Of note, increasing the amount of Vaseline or repeating the application every day allows longer experimental windows. For subsequent data analysis the animals were divided into four different categories (A–D) according to their posture/position (Fig. 1A) and iBAT temperature or maximum iBAT temperature (highest temperature measured in one session) were determined using the software FLIR Tools Version 5 or 6 (FLIR, Sweden, https://www.flir.de/products/flir-tools/).

Infrared videos of animals without (−) and with (+) Vaseline were recorded with a 5s interval using a VarioCAM hr head camera (InfraTec, with a thermal sensitivity of < 0.05 °C and an accuracy of 2% max 1.5 °C, Germany). Measurements were carried out on fasted (2 h) animals in a temperature-controlled chamber at 23 °C. Vaseline (~ 10 mg) was applied right before the experimental start, the cotton nestlets were removed and the animals were placed back in their home cage to reduce stress. Baseline iBAT temperature and maximum iBAT temperature before injection were calculated in a 10-min period of reduced activity. Since this period was not always immediately prior to injection, a continuous time axis was omitted in Fig. 4E. The thermogenic response to norepinephrine (NE) was determined by injecting the animals (N = 6) subcutaneously with 1 mg/kg arterenol (Sanofi, Germany)^16,17^, afterwards they were filmed for another 60 min. Maximum iBAT temperature was scored within the first 30 min after NE injection using the IRBIS3 software (InfraTec, Germany, https://www.infratec.de/thermografie/thermografie-software/irbis3/).

Rectal body temperature was measured with a thermal probe (BAT-12, Physitemp, USA) and taken as an estimate of core body temperature.

### Indirect calorimetry

To detect possible changes in metabolic rate caused by Vaseline application or the resulting decreased thermal insulation of the fur (resp. increased heat conductance), 3 months old C57BL/6NCr wild-type mice (N = 6) were measured via indirect calorimetry using an open respirometry system (TSE PhenoMaster, TSE Systems, Germany). First, the animals were acclimated to a temperature-controlled chamber (23 ± 0.5 °C) and energy expenditure, food and water intake, respiratory quotient (RQ = carbon dioxide produced/oxygen consumed) and activity was measured simultaneously at 20-min intervals. After the animals got used to the climate chamber Vaseline was applied to one group of animals (9:00 a.m., (+) Vaseline), whereas the control group received a sham treatment (swap without Vaseline, (−) Vaseline). Daily energy expenditure (DEE, kJ/day) was calculated over 24 h using the metabolic rate (VO_2_ (ml O_2_/h)), the RQ and the caloric equivalents given by^18^ (heat production (HP, mW) = (4.44 + 1.43 * RQ) * VO_2_ (ml O_2_/h)). Furthermore, resting metabolic rate at 23 °C (RMR) was determined in the inactive phase of mice (lights on) and over a 1-h interval of consistently low oxygen consumption rates and without physical activity.

Resting metabolic rate at thermoneutrality (30 °C, basal metabolic rate = BMR) was calculated from the lowest mean oxygen consumption over a period of 1h in a postabsorptive (fasted for 6 h) and non-moving animal after Vaseline application (~ 10 mg). Data were analyzed with TSE PhenoMaster software V6.5.3 (TSE Systems, Germany, https://www.tse-systems.com/product-details/phenomaster).

### Thermal hot bridge analysis

Skin samples still containing fur were dissected from the interscapular area of C57BL/6NCr wild-type mice (N = 6) and analyzed using a transient hot bridge instrument (Linseis System THB; Gammadata Instruments, Sweden) to validate whether Vaseline changes the conductivity of the fur and increases heat loss over the skin surface. One group of animals received the Vaseline (~ 10 mg) 24 h before the measurement, whereas the skin of the other group was first measured without Vaseline and then directly after Vaseline application (~ 150 mg). A Kapton sensor type A was used to heat the sample, and the resulting temperature change was measured to determine heat conductivity (W/(m*K), reference range skin = 0.21–0.41)^8^ and diffusivity (mm^2^/s). Heat capacity (J/(gK), calculated assuming ρ = 1.0 g/cm^3^) was calculated subsequently.

### Western Blot analysis

To determine whether Vaseline treatment changes the molecular signature of brown adipocytes, iBAT samples of animals without and 24 h after Vaseline (N = 6) treatment were analyzed. For protein isolation snap-frozen iBAT samples were used and protein concentrations were determined using Pierce BCA Kit (Thermo Fisher Scientific, Germany). For immunological detection 20 µg of iBAT (not pre-heated to 98 °C) were separated on a 12% SDS polyacrylamide gel and transferred onto a polyvinylidene fluoride membrane (Merck Millipore, Germany). Membranes were probed with a rabbit anti-UCP1 polyclonal antibody (1:10,000 dilution, previously used in^19^) or a mouse anti-OxPhos antibody cocktail (Cat#45-8099, Invitrogen, 1:2000 dilution) followed by a peroxidase-conjugated secondary antibody (goat anti-rabbit-IgG HRP-conj. at 1:5000 dilution, Cat#P0448, DAKO, or goat anti-mouse-IgG HRP-conj. at 1:5000 dilution, Cat#P0447, DAKO (Denmark)). Then antigens were visualized using an ECL Plus Western blotting detection system (Chemi Doc Touch, BioRad, Germany) and band intensity was quantified using Image Lab software 6.1 (BioRad, Germany, https://www.bio-rad.com/de-de/product/image-lab-software). Data were normalized to total protein load and controls (without (−) Vaseline).

### Quantitative real-time PCR (qPCR)

RNA isolation was performed following the manufacturer’s instructions using RNeasy Lipid Tissue Mini Kit (QIAGEN, Germany, N = 6). Subsequent cDNA synthesis was performed using the Molecular Biology RevertAid Strand cDNA Kit (Thermo Fisher Scientific, Germany) with anchored oligo (dT)18 primers. Quantstudio Applied Biosystems (Thermo Fisher Scientific, Germany) and SYBR green PCR master mix (+Rox, Roche, Germany) were used for qPCR analysis. The most stable reference genes were determined with geNorm version 3.4^20^ and gene expression levels were normalized with *hypoxanthine phosphoribosyltransferase* (*Hprt)* and *cyclophilin D (Cyclo)*. The expression of *uncoupling protein 1* (*Ucp1*), *β3-adrenergic receptor* (*Adrb3*) and *deiodinase 2* (*Dio2*) were measured and results were calculated using the ∆∆CT method, and standard curves were recorded to correct for PCR efficiency. Primer sequences are available on request.

### Statistical analysis

GraphPad Prism 7 software was used to analyze the data (GraphPad Software, San Diego, USA). Values are represented as mean, box plot (min. to max.), mean ± SD (in vivo experiments) or mean ± SEM (molecular analysis), as indicated in figure legends. Statistical testing was performed using Student’s t-test with Welch’s correction, paired Student’s t-test, 1-way or 1-way repeated measures ANOVA with Tukey’s post-hoc test, 2-way or 2-way repeated measures ANOVA with Bonferroni’s post-hoc test where indicated. *p* values of less than 0.05 were considered significant, and the respective levels of significance are *p < 0.05, **p < 0.01, ***p < 0.001.

## Results

### Vaseline enables more reproducible iBAT IR-pictures

The quality and reproducibility of iBAT IR-pictures taken from freely moving mice is highly dependent on (a) the activity level of the animal, (b) the quality of the fur and (c) the time it takes an experimenter to photograph the animal in the correct position (Fig. 1A). All three criteria (a, b and c) influence each other and can lead to pronounced differences in the comparability of infrared (IR) pictures. For example, anxious animals show a less explorative behavior and due to that might spend more time sitting in a curled up position during an experimental session (Fig. 1A–C, mouse position A). However, in this position it is not possible to photograph the animal’s skin through the fur and accordingly iBAT temperature is underestimated (Fig. 1D,F, mouse position A compared to mouse position D). Maximum iBAT temperature of animals sitting in a curled up position or grooming themselves was only 34.41 °C ± 1.12 °C, whereas animals that stretch forward at the right angle, allowing a direct view on the skin, reached a maximum iBAT temperature of 38.69 °C ± 0.20 °C. This direct view on the animal’s skin was responsible for a 2 °C increase in maximum iBAT temperature (mouse position C compared to mouse position D), emphasizing the importance of the fur for iBAT IR-thermography.

Based on these results, our aim was to develop a method that minimizes the disturbing effect of the fur on iBAT IR-temperature without influencing the conductivity for a long time (like shaving) and to establish a rapid and minimal invasive method for quantifying BAT thermogenesis using infrared thermography, which we termed Precise Unravelment of Non-shivering thermoKinetics (PUNK) protocol. Brushing up the fur with a minimum amount of Vaseline (~ 10 mg) indeed minimized the effect of the fur on iBAT temperature and led to more stable and reproducible measurements (Fig. 1C,E). Additional, the position of the mouse was no longer critical for the recording of maximum iBAT temperature (Fig. 1G). Only between animals in a curled up or stretched position a difference was still detectable, however, this difference decreased to 0.5 °C compared to > 4 °C without Vaseline.

### No thermogenic side-effects of Vaseline application

**Figure 2:**
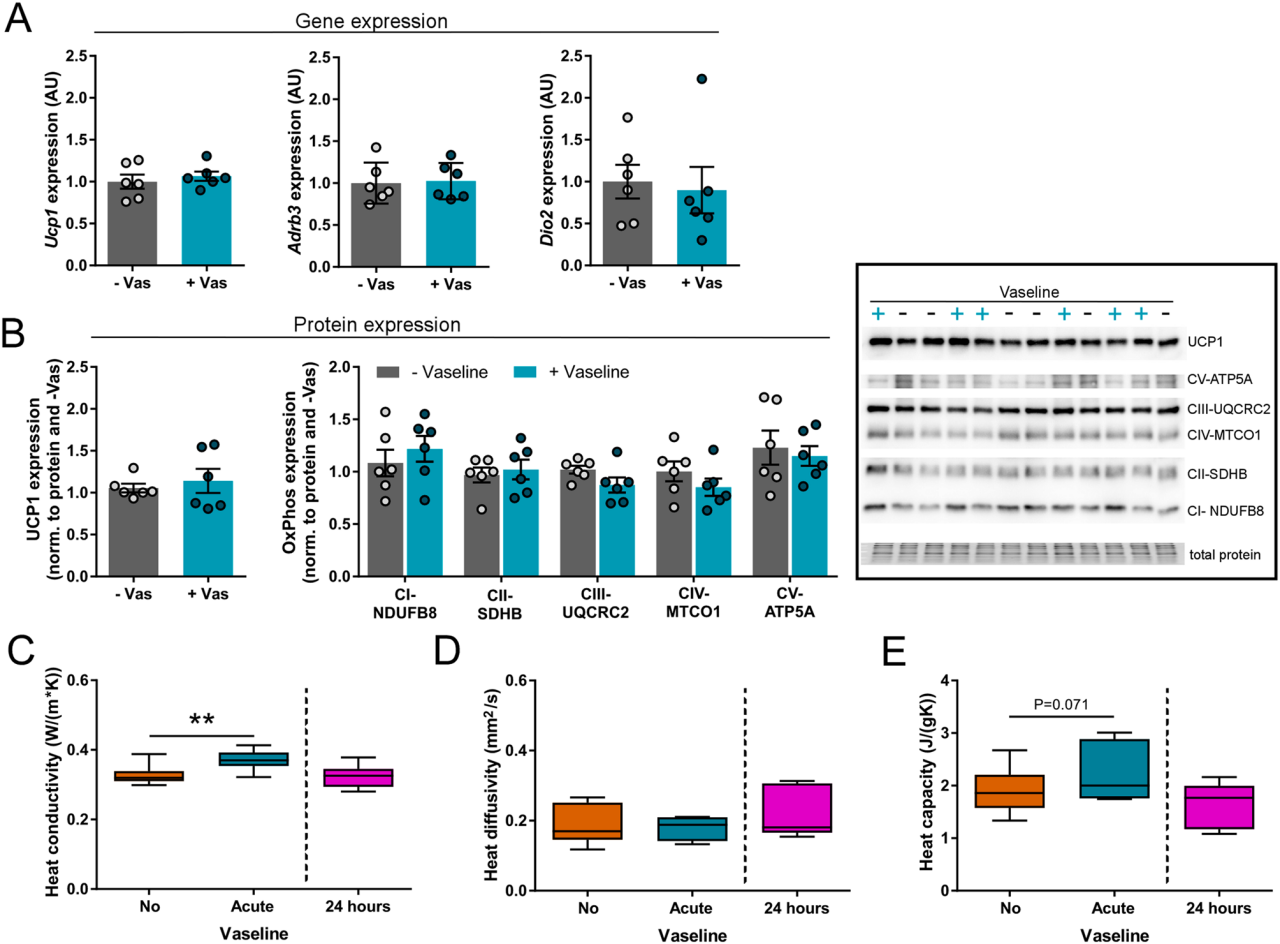
The thermogenic profile of BAT cells is not altered by Vaseline application. (**A**–**C**) The expression of important thermogenic genes (*uncoupling protein 1* (*Ucp1*), *β3-adrenergic receptor* (*Adrb3*), *deiodinase 2* (*Dio2*) and proteins (UCP1 and OxPhos) was unchanged 24 h after Vaseline treatment. (**C**–**E**) Furthermore, heat conductivity, heat diffusivity and heat capacity of the skin were normal 24 h after application, whereas acute application of Vaseline resulted in an increased heat conductivity. N = 6, data are presented as box plot (min. to max., **C**–**E**) or mean ± SEM (**A**,**B**), *p < 0.05, **p < 0.01, ***p < 0.001 (Student’s t-test with Welch’s correction (**A**,**B** (UCP1/OxPhos blot), **C**–**E** (groups no and 24 h Vaseline) or paired Student’s t-test (C – E (groups no and acute Vaseline)).

We next determined whether Vaseline leads to alterations in the thermogenic program of the brown adipocytes. Therefore, we measured the expression of thermogenic genes (*uncoupling protein 1* (*Ucp1*), *β3-adrenergic receptor* (*Adrb3*) and *deiodinase 2* (*Dio2*)) 24 h after Vaseline application as well as UCP1 and OxPhos protein as a read-out for thermogenesis and mitochondrial power (Fig. 2A,B, Supplementary Fig. 1). No difference been animals without (−) and with ( +) Vaseline could be detected, which was in line with no alterations in heat conductivity, heat diffusivity and heat capacity of skin samples containing fur (Fig. 2C–E). Only the acute application of high amounts of Vaseline led to a significant elevation in heat conductivity and a slight increase in heat capacity compared to the same skin sample without Vaseline (Fig. 2C,E).

### No metabolic side-effects of Vaseline application

**Figure 3:**
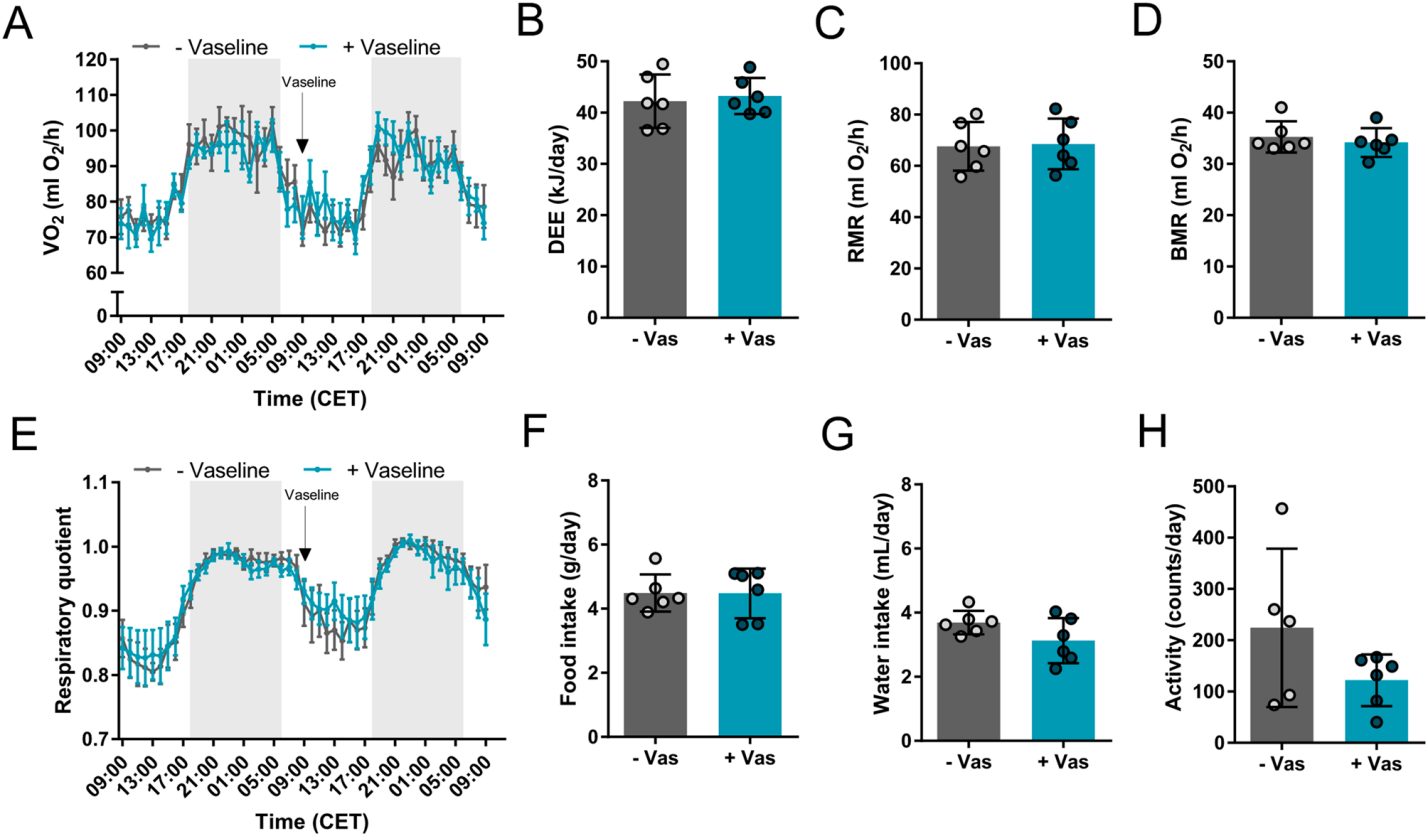
Vaseline application does not change energy expenditure. After acclimation to the climate chamber, Vaseline was applied to one group of animals (9:00 am, + Vaseline), whereas the other group received a sham treatment. (**A**) Oxygen consumption, as well as (**B**) daily energy expenditure (DEE), (**C**) resting metabolic rate at 23 °C (RMR) and resting metabolic rate at thermoneutrality (30 °C, basal metabolic rate, BMR) were not altered by Vaseline application. Furthermore, decreasing the thermal insulation of the fur did not result in changes in (**E**) fuel utilization (respiratory quotient, RQ), (**F**) food intake, (**G**) water intake or H) activity. N = 6, data are presented as mean ± SD (**B**–**D**,**F**–**H**) or mean ± SEM (**A**,**E**). *p < 0.05, **p < 0.01, ***p < 0.001 (Student’s t-test with Welch’s correction (**B**–**D**,**F**–**H**), 2-way repeated measures ANOVA with Bonferroni’s post-hoc test (**A**,**E**)).

Vaseline is a metabolically inert substance that is not expected to alter metabolism when ingested in small amounts during grooming behavior or licking the fur. However, as Vaseline changes the insulating characteristics of the fur when acutely applied in high amounts (~ 150 mg, Fig. 2C–E), it might be possible that Vaseline increases heat loss and thus daily energy expenditure (DEE). In order to exclude long-term metabolic side-effects of Vaseline application, we measured the DEE of animals without (−) and with ( +) Vaseline (~ 10 mg) for 24 h using indirect calorimetry (Fig. 3). As expected, Vaseline had no effect on oxygen consumption (VO_2_), DEE, RMR at 23 °C and 30 °C (BMR) or the respiratory quotient (Fig. 3A–E). Furthermore, food and water intake as well as activity were unchanged (Fig. 3F–H), demonstrating no acute or long-term effect of Vaseline on whole body metabolism.

### Vaseline allows a precise unravelment of non-shivering thermokinetics

**Figure 4:**
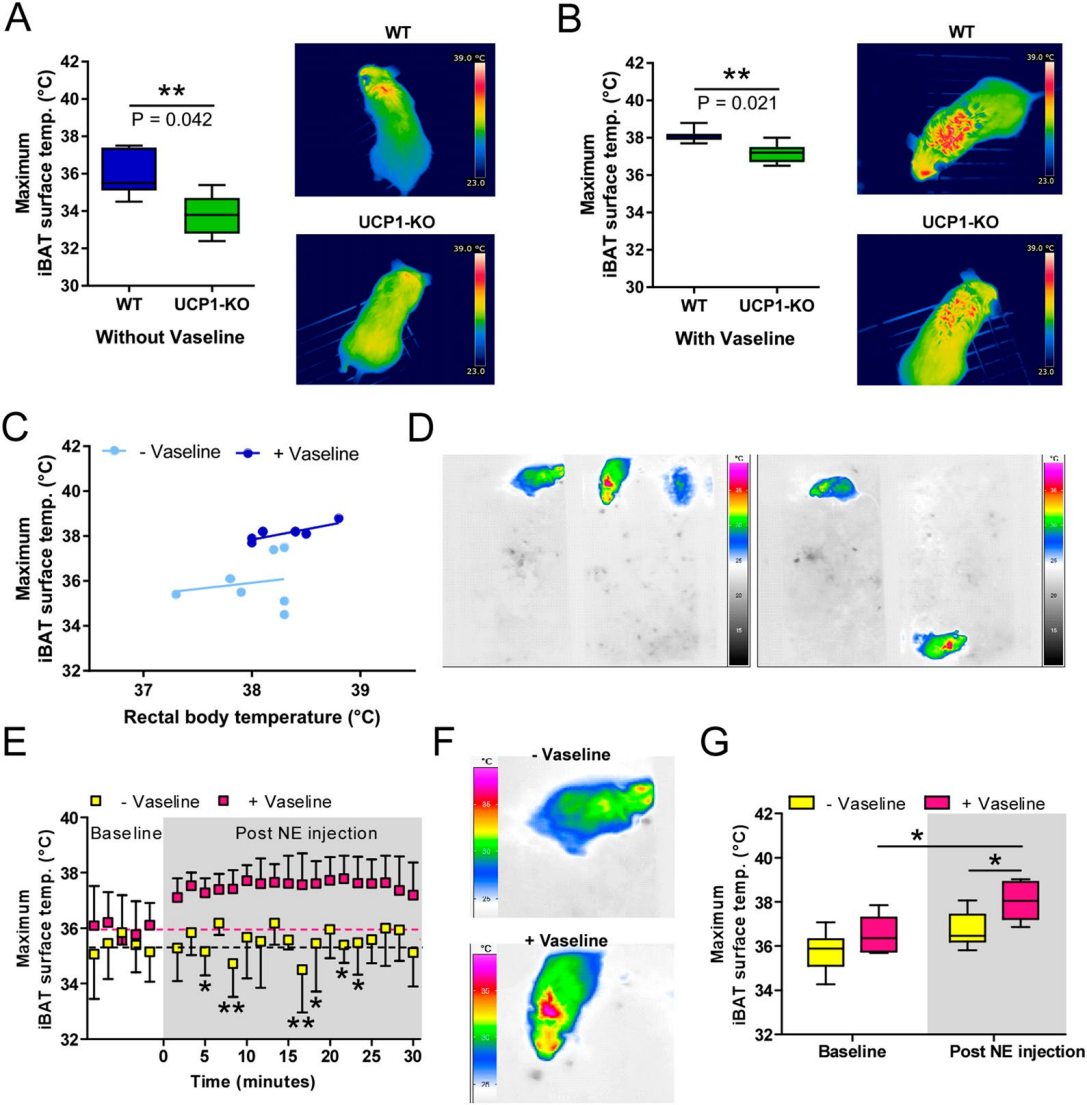
Vaseline makes the quantification of non-shivering thermokinetics more precise. (**A**,**B**) Quantification and representative infrared pictures of wild-type (WT) and UCP1-KO mice without (−) and with (+) Vaseline, showing higher maximum iBAT temperatures and more stable results after Vaseline application (N = 7). (**C**) Correlation of maximum iBAT surface temperature and rectal body temperature of wild-type mice with and without Vaseline (N = 7). (**D**) Representative infrared pictures from video recordings after norepinephrine (NE) injection (1 mg/kg, left mouse: (−) Vaseline, right mouse: (+) Vaseline). (**E**) Maximum iBAT temperature of wild-type mice before and post NE injection. Of note, baseline measurements were not always obtained immediately before the NE injection, therefore a continuous time-axis before the injection was omitted. (**F**) Representative picture of a mouse without (−) Vaseline and with (+) Vaseline at the peak after NE injection. (**G**) Quantification of the maximum iBAT temperature of wild-type mice after before and post NE injection, showing only in the (+) Vaseline group a significant increase in iBAT temperature (N = 6). Data are presented as box plot (min. to max., **A**,**B**,**G**) or mean ± SD (**E**), *p < 0.05, **p < 0.01, ***p < 0.001 (Student’s t-test with Welch’s correction (**A**,**B**) or 2-way repeated measures ANOVA with Bonferroni’s post-hoc test (**E**,**G**)).

To validate whether the PUNK protocol facilitates a more precise detection of differences in iBAT activity compared to IR pictures taken without Vaseline, we analyzed iBAT temperature of UCP1-KO mice (no active BAT) and wild-type (WT) mice after NE injection (fully activated BAT, Fig. 4). Analysis of iBAT surface temperature in UCP1-KO mice revealed lower maximum iBAT temperatures without Vaseline (only mouse position D) and with Vaseline (mouse position C and D), but the absolute iBAT temperature was higher after Vaseline application (Fig. 4A,B). Most remarkably, the PUNK protocol increased the effect size (without Vaseline: dz = 1.913 ; with Vaseline: dz = 2.169), by strongly reducing the variability within the groups (SD without Vaseline: ± 1.14 (WT) and ± 1.05 (UCP1-KO); SD with Vaseline: ± 0.34 (WT) and ± 0.50 (UCP1-KO)). Furthermore, maximum iBAT surface temperature of wild-type mice showed a stronger correlation with rectal body temperature after Vaseline application (Fig. 4C, R^2^ without Vaseline: 0.03274 and with Vaseline: 0.698).

In the next step, we analyzed infrared videos with high time resolution taken before and after NE injection to test the suitability of our protocol for longitudinal studies and automated picture analyses (Fig. 4D,F, Supplemental Video 1). In line with previous studies^21^, the increase in maximum iBAT temperature (baseline vs. post NE injection) was barely visible in animals without Vaseline (Fig. 4E,G), which might be attributed to the fact that the animals often show a reduced locomotion after NE injection and even lie down flat to release heat thereby preventing a direct line of sight though the fur to the skin. Although, maximum iBAT temperature increased from 35.8 °C at baseline to 36.5 °C post NE injection, this effect was not statistically significant. Most remarkably, the measured increase in maximum iBAT temperature post NE injection was much more pronounced in the Vaseline group, clearly unravelling the expected elevation in iBAT thermogenesis (from 36.5 °C (baseline) to 38.0 °C (post NE injection) and reaching statistical significance (Fig. 4G, Supplemental Video 1).

## Discussion

Infrared pictures of the hairy or shaved skin covering the inter-scapular area are commonly taken to measure iBAT thermogenesis^5,6,8,10,11,13^. However, obtaining measurements of sufficient quality is challenging and their interpretation can be difficult. Not only is the right position of the mouse critical important to be able to photograph the skin through the fur, but also the condition of the fur itself. For example, different KO mouse models used in BAT/obesity research have less or more greasy fur and thus are not directly comparable to their wild type litter mates^22^. In addition, during aging mice show a reduction in grooming behaviour, as a result their fur gets fattier and exposes more skin at the iBAT region, which makes it difficult to interpret results obtained from mice of different ages. The same applies to studies on rodents fed a high fat diet, with reduced locomotion (e.g. obesity models) or mental disorders, leading to confounding effects in IR thermography of BAT thermogenesis.

To circumvent these problems, several studies employed shaving their animals to eliminate the possibly disturbing effect of the fur^11,12,23–25^. However, shaving creates “thermal windows” and given that the thermal conductivity of the insulating surface (fur, skin and the subcutaneous fat) is the most notable physiological factor affecting heat flow between an animal and its environment^9^, shaved mice perceive their environment as much colder (for instance nude mice perceive 20 °C as 10 °C)^22^. Subsequently, BAT is recruited, browning of WAT is induced and metabolism is increased by up to 50% (depending on the size of the shaved area^22,26,27^. Thus, shaving the animals can be considered equivalent to exposing an animal to a colder temperature and cooling the BAT area may even directly spark changes in adipose tissue given that the tissue has heat sensing capabilities^15^. In addition, shaving usually requires anaesthesia, can irritate the skin leading to inflammation (higher blood flow, higher temperature)^23^, or needs to be applied repeatedly for longitudinal studies, given that the fur grows back rapidly^28^.

As an alternative to shaving, anaesthesia can be used to improve the reproducibility of IR thermography measurements and to reduce the number of animals required^6^. However, anaesthesia is invasive and body temperature needs to be maintained to avoid effects of body cooling. Thus, performing long-term measurements or longitudinal studies is almost impossible, especially when considering that volatile anaesthetics such as halothane or isoflurane inhibit BAT activity^29,30^. Consequently, the absolute magnitude of BAT temperature can never be obtained from anaesthetized animals, and techniques requiring anaesthesia such as ^18^F-FDG PET/CT scans will suffer from similar problems. Moreover, it has been shown that in BAT ^18^F-FDG uptake can occur independently of UCP1 function^31^, which can therefore only provide a rough approximation of true BAT thermogenesis In contrast, implanted probes for precise determination of iBAT temperature, which are fixed close to the Sulzer’s vein, allow the recording of absolute iBAT temperature (reviewed by^9^). However, this method is rather invasive due to the required surgery and thus more complex and error-prone, and often not feasible for larger cohorts of animals. The most critical point of infrared thermography is the influence of ambient temperature on surface temperature thermography^32,33^. Skin temperature is strongly dependent on ambient temperature and control over ambient temperature is absolutely required to obtain reliable and comparable results.

Consequently, our PUNK protocol provides an enormous advantage over previously used infrared techniques, as the animals do not need to be anesthetised or shaved. Yet it allows the performance of highly robust and reproducible measurements, increasing the statistical power of obtained results and thus reducing the required number of animals. Therefore, the PUNK protocol can be used as a complementing technique to the common gold standard of measuring NST capacity, namely indirect calorimetry^34^, as it facilitates an automated analysis of IR videos. With Vaseline the BAT is consistently the hottest spot in the IR pictures over the entire time frame of the experiment, thus eliminating the need for manually scoring large numbers of IR pictures. Importantly, good science goes always hand in hand with good animal welfare. Whilst infrared thermography is commonly classified as a non-invasive method by national animal welfare committees and therefore often does not require an animal ethical application, add-on procedures like shaving and anaesthetizing are invasive and cause distress to the animals. Therefore, in addition to providing data of higher quality, our PUNK protocol also provides a simple way to adhere to the 3R’s guiding principle for a more ethical use of animals in BAT research.

## Supplementary Information

Supplementary Information 1.

Supplementary Video 1.

## Data Availability

The original data are available upon reasonable request from the corresponding author.

## References

[1] Bartelt A (2011). Brown adipose tissue activity controls triglyceride clearance. Nat. Med..

[2] Kajimura S, Spiegelman BM, Seale P (2015). Brown and beige fat: physiological roles beyond heat generation. Cell Metab..

[3] Matsushita M (2014). Impact of brown adipose tissue on body fatness and glucose metabolism in healthy humans. Int. J. Obes..

[4] Nedergaard J, Cannon B (2013). UCP1 mRNA does not produce heat. Biochim. Biophys. Acta.

[5] Johann K (2019). Thyroid-hormone-induced browning of white adipose tissue does not contribute to thermogenesis and glucose consumption. Cell Rep..

[6] Crane JD, Mottillo EP, Farncombe TH, Morrison KM, Steinberg GR (2014). A standardized infrared imaging technique that specifically detects UCP1-mediated thermogenesis invivo. Mol. Metab..

[7] Sampath SC, Sampath SC, Bredella MA, Cypess AM, Torriani M (2016). Imaging of brown adipose tissue: state of the art. Radiology.

[8] Warner A (2013). Inappropriate heat dissipation ignites brown fat thermogenesis in mice with a mutant thyroid hormone receptor α1. Proc. Natl. Acad. Sci. USA.

[9] Meyer CW, Ootsuka Y, Romanovsky AA (2017). Body temperature measurements for metabolic phenotyping in mice. Front. Physiol..

[10] Oelkrug R (2017). Dwarfism and insulin resistance in male offspring caused by α1-adrenergic antagonism during pregnancy. Mol. Metab..

[11] Åkesson L (2007). Decreased core temperature and increased *β*_3_ -adrenergic sensitivity in diabetes-prone BB rats. Diabetes Technol. Ther..

[12] Marks A, Vianna DML, Carrive P (2009). Nonshivering thermogenesis without interscapular brown adipose tissue involvement during conditioned fear in the rat. Am. J. Physiol. Integr. Comp. Physiol..

[13] Whittle AJ (2012). BMP8B increases brown adipose tissue thermogenesis through both central and peripheral actions. Cell.

[14] Kutyavin VI, Chawla A (2019). BCL6 regulates brown adipocyte dormancy to maintain thermogenic reserve and fitness. Proc. Natl. Acad. Sci. USA.

[15] Ye L (2013). Fat cells directly sense temperature to activate thermogenesis. Proc. Natl. Acad. Sci. USA.

[16] Dicker A, Cannon B, Nedergaard J (1995). Cold acclimation-recruited nonshivering thermogenesis: the Syrian hamster is not an exception. Am. J. Physiol. Regul. Integr. Comp. Physiol..

[17] Oelkrug R, Polymeropoulos ET, Jastroch M (2015). Brown adipose tissue: physiological function and evolutionary significance. J. Comp. Physiol. B..

[18] Heldmaier G (1975). Metabolic and thermoregulatory responses to heat and cold in the Djungarian hamster Phodopus sungorus. J. Comp. Physiol..

[19] Jastroch M (2012). Expression of uncoupling proteins in a mammalian cell culture system (HEK293) and assessment of their protein function. Methods Mol. Biol..

[20] Vandesompele J (2002). Accurate normalization of real-time quantitative RT-PCR data by geometric averaging of multiple internal control genes. Genome Biol..

[21] Jackson DM, Hambly C, Trayhurn P, Speakman JR (2001). Can non-shivering thermogenesis in brown adipose tissue following NA injection be quantified by changes in overlying surface temperatures using infrared thermography?. J. Therm. Biol..

[22] Nedergaard J, Cannon B (2014). The browning of white adipose tissue: some burning issues. Cell Metab..

[23] Carter EA (2011). Association of heat production with 18F-FDG accumulation in murine brown adipose tissue after stress. J. Nucl. Med..

[24] Gachkar S (2017). 3-Iodothyronamine induces tail vasodilation through central action in male mice. Endocrinology.

[25] Dore R (2017). The thermogenic effect of nesfatin-1 requires recruitment of the melanocortin system. J. Endocrinol..

[26] Zhao ZJ, Cao J (2009). Effect of fur removal on the thermal conductance and energy budget in lactating Swiss mice. J. Exp. Biol..

[27] Szafranska PA (2014). Shaving increases daily energy expenditure in free-living root voles. J. Exp. Biol..

[28] Müller-Röver S (2001). A comprehensive guide for the accurate classification of murine hair follicles in distinct hair cycle stages. J. Investig. Dermatol..

[29] Ohlson KBE, Mohell N, Cannon B, Lindahl SGE, Nedergaard J (1994). Thermogenesis in brown adipocytes is inhibited by volatile anesthetic agents: a factor contributing to hypothermia in infants?. Anesthesiology.

[30] Ohlson KBE, Lindahl SGE, Cannon B, Nedergaard J (2003). Thermogenesis inhibition in brown adipocytes is a specific property of volatile anesthetics. Anesthesiology.

[31] Hankir MK (2017). Dissociation between brown adipose tissue 18F-FDG uptake and thermogenesis in uncoupling protein 1-deficient mice. J. Nucl. Med..

[32] Tattersall GJ (2016). Infrared thermography: a non-invasive window into thermal physiology. Comp. Biochem. Physiol. Part A Mol. Integr. Physiol..

[33] Hodges MR (2008). Defects in breathing and thermoregulation in mice with near-complete absence of central serotonin neurons. J. Neurosci..

[34] Oelkrug R, Polymeropoulos ET, Jastroch M (2015). Brown adipose tissue: physiological function and evolutionary significance. J. Comp. Physiol. B.

