# Supplementary material for: An improved method for the precise unravelment of non-shivering brown fat thermokinetics
Source: Sci Rep. 2021 Feb 26;11:4799. doi: 10.1038/s41598-021-84200-1 (PMC7910537; doi:10.1038/s41598-021-84200-1)
Supplement: Supplementary file 1 — Supplementary Information 1. [file 41598_2021_84200_MOESM1_ESM.pdf]

An improved method for the precise unravelment of non-shivering brown fat thermokinetics

Rebecca Oelkrug[a,*] and Jens Mittag[a]

Supplementary Video 1

The infrared video shows baseline and response to a single injection of norepinephrine (1mg/kg s.c.) comparing mice with (right) and without (left) Vaseline to brush of the fur above the brown adipose tissue.

Supplementary Figure 1

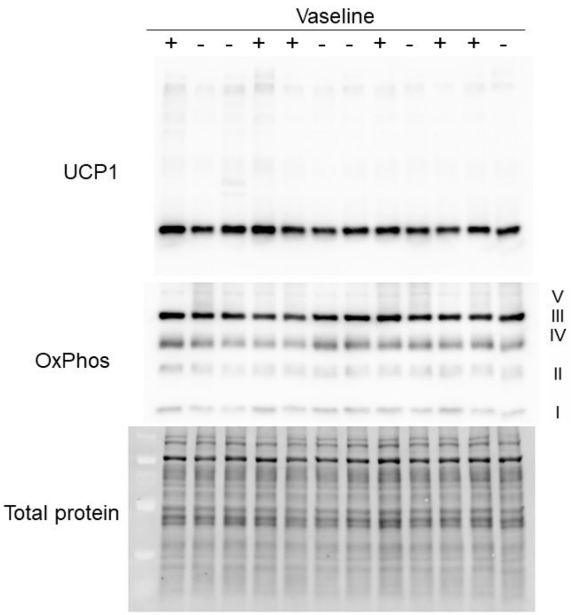

Full Western Blot membranes of the data depicted in Figure 2A and B.
